# Simultaneous Qualitative and Quantitative Analysis of Multiple Chemical Constituents in YiQiFuMai Injection by Ultra-Fast Liquid Chromatography Coupled with Ion Trap Time-of-Flight Mass Spectrometry

**DOI:** 10.3390/molecules21050640

**Published:** 2016-05-18

**Authors:** Chunhua Liu, Aichun Ju, Dazheng Zhou, Dekun Li, Junping Kou, Boyang Yu, Jin Qi

**Affiliations:** 1 Jiangsu Key Laboratory of TCM Evaluation and Translational Research, China Pharmaceutical University, Nanjing 211198, China; liu_hua139@126.com (C.L.); junpingkou@163.com (J.K.); 2 Department of Technology Development, TianJin Tasly Pride Pharmaceutical Co., Ltd., Tianjin 300410, China; juach@tasly.com (A.J.); zhoudz@tasly.com (D.Z.); lidekun@tasly.com (D.L.); 3 State Key Laboratory of Natural Medicines, China Pharmaceutical University, Nanjing 210009, China

**Keywords:** YiQiFuMai injection, UFLC-IT-TOF/MS, ophiopogonins, identification, quantification

## Abstract

YiQiFuMai injection (YQFM) is a modern lyophilized powder preparation derived from the traditional Chinese medicine Sheng-mai san (SMS) used for treating cardiovascular diseases, such as chronic heart failure. However, its chemical composition has not been fully elucidated, particularly for the preparation derived from *Ophiopogon japonicus*. This study aimed to establish a systematic and reliable method to quickly and simultaneously analyze the chemical constituents in YQFM by ultra-fast liquid chromatography coupled with ion trap time-of-flight mass spectrometry (UFLC-IT-TOF/MS). Sixty-five compounds in YQFM were tentatively identified by comparison with reference substances or literature data. Furthermore, twenty-one compounds, including three ophiopogonins, fifteen ginsenosides and three lignans were quantified by UFLC-IT-TOF/MS. Notably, this is the first determination of steroidal saponins from *O. japonicus* in YQFM. The relative standard deviations (RSDs) of intra- and inter-day precision, reproducibility and stability were <4.9% and all analytes showed good linearity (*R*^2^ ≥ 0.9952) and acceptable recovery of 91.8%–104.2% (RSD ≤ 5.4%), indicating that the methods were reliable. These methods were successfully applied to quantitative analysis of ten batches of YQFM. The developed approach can provide useful and comprehensive information for quality control, further mechanistic studies *in vivo* and clinical application of YQFM.

## 1. Introduction

YiQiFuMai injection (YQFM) is a modern lyophilized powder preparation based on the traditional Chinese medicine (TCM) formula Sheng-mai san (SMS), composed of three herbs: *Ginseng Radix* et *Rhizoma Rubra* (red ginseng) from the root of *Panax ginseng* C.A. Mey., *Ophiopogonis Radix* from the root of *Ophiopogon japonicus* (L. f) Ker-Gawl. and *Schisandrae chinensis Fructus* from the fruits of *Schisandra chinensis* (Turcz.) Baill [1,2,3]. Both SMS and YQFM have been used in the treatment of cardiovascular diseases for many years [4,5], and considerable clinical evidence has confirmed that YQFM has superior clinical efficacy and fewer side effects in the treatment of heart diseases, such as chronic heart failure and hypertrophic obstructive cardiomyopathy [5,6].

A large number of literature studies have shown that ginsenosides, lignans and ophiopogonins are the major bioactive constituents in SMS and some of its modern preparations [1,2,7,8,9]. Many researchers have studied the ginsenosides and lignans in YQFM or other Sheng-mai preparations using HPLC-UV/MS or near infrared spectroscopy [10,11,12,13,14], but few of the studies have focused on analysis of ophiopogonins in YQFM. *O. japonicus*, which contains ophiopogonins, is an essential component of SMS, conferring better biological activity in terms of cardiovascular diseases [15,16,17,18]. However, there have been no recent reports on the determination of ophiopogonins in YQFM. Consequently, the global chemical composition of YQFM has remained obscure, impeding process quality control and mechanistic studies.

In this study, ophiopogonins were both qualitatively and quantitatively analyzed using ultra-fast liquid chromatography coupled with ion trap time-of-flight mass spectrometry (UFLC-IT-TOF/MS). This system has been established as a reliable method for structural and quantitative analysis, integrating the capabilities of IT and TOF MS with LC for efficient separation in a single instrument [19,20,21,22,23,24]. Sixty-five compounds were identified from YQFM by UFLC-IT-TOF/MS, including the first identification of seven compounds from *O. japonicus*, comprising four steroidal saponins, one borneol pyranoside and two flavonoids. Additionally, three ophiopogonins, fifteen ginsenosides and three lignans in ten batches of YQFM were measured using validated quantitative analysis methods. The present study provides new information and supporting data for the quality control and clinical application of YQFM.

## 2. Results and Discussion

### 2.1. Optimization of Chromatographic Conditions

Solid phase extraction (SPE) was used to remove the abundant saccharides present in YQFM to enable better analysis of non-saccharide small molecules. To achieve optimal separation and strong ion signals, the chromatographic conditions, including column, mobile phase and column temperature, were optimized. An ACQUITY UPLC HSS T3 column (100 mm × 2.1 mm, 1.8 µm, Waters, Milford, MA, USA) was chosen for high resolution after comparison with XB-C18 100A (Phenomenex, Torrance, CA, USA) and ZORBAX SB-C18 (Agilent, Palo Alto, CA, USA) columns. Moreover, different linear gradients of mobile phase A (water with acetic acid (0.05%, *v*/*v*) or formic acid (0.05% or 0.1%, *v*/*v*)) and mobile phase B (acetonitrile with acetic acid (0.05%, *v*/*v*) or formic acid (0.05% or 0.1%, *v*/*v*) were tested. Ultimately, water with formic acid (0.05%, *v*/*v*) and acetonitrile were chosen as the eluent since it provided an appropriate intensity of the base peak chromatogram (BPC) (Figure S1 in the Supplementary Materials). The effect of different accumulation times on the BPC were investigated, and 100 ms was selected for subsequent analyses (Figure S2).

### 2.2. Identification of Chemical Constituents in YQFM

**Figure 1:**
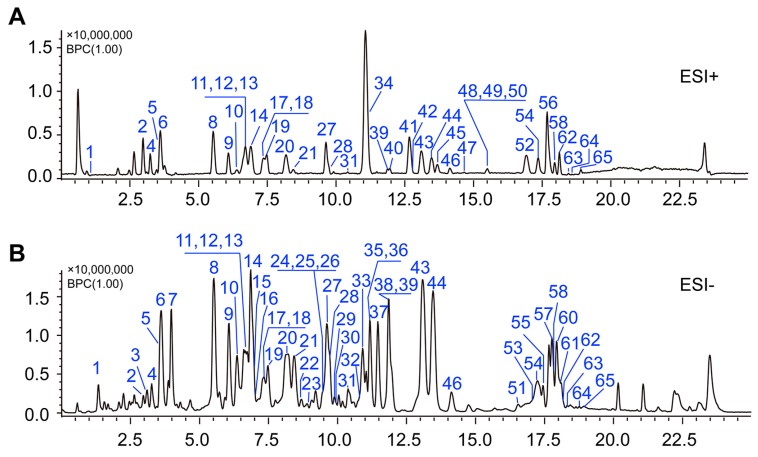
The base peak chromatograms (BPC) of YiQiFuMai injection by UFLC-IT-TOF/MS in negative (**A**) and positive ion mode (**B**).

Representative MS^1^ BPC of YQFM using the UFLC-IT-TOF/MS method after optimization of the chromatographic conditions are shown in Figure 1A,B. In this study, some compounds were identified by comparison with reference standards and others were tentatively characterized based on their retention times and MS spectra, referring to the literature [1,13,25,26,27,28,29,30,31,32,33,34,35,36]. Ultimately, a total of sixty-five compounds were identified or tentatively characterized, of which forty-two compounds were from *P. ginseng*, sixteen from *S. chinensis*, and seven compounds from *O. japonicas*, including four steroidal saponins, one borneol pyranoside and two flavonoids (Table S1).

#### 2.2.1. Identification of Compounds in *O. japonicus*

**Figure 2:**
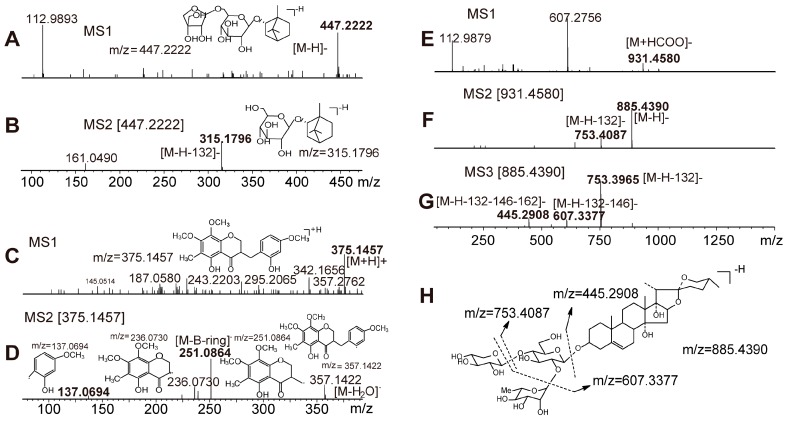
MS spectra of L-borneol-7-*O*-[β-d-apiofuranosyl(1→6)]-β-d-glucopyranoside, 5,2′-dihydroxy-7,8,4′-trimethoxy-6-methylhomoisoflavanone and ophiopojaponin C, and a proposed fragmentation pathway. ((**A**): MS^1^ spectrum of L-borneol-7-*O*-[β-d-apiofuranosyl(1→6)]-β-d-glucopyranoside under negative mode; (**B**): MS^2^ spectrum of L-borneol-7-*O*-[β-d-apiofuranosyl(1→6)]-β-d-glucopyranoside under negative mode; (**C**): MS^1^ spectrum of 5,2′-dihydroxy-7,8,4′-trimethoxy-6-methylhomoisoflavanone under positive mode; (**D**): MS^2^ spectra of 5,2′-dihydroxy-7,8,4′-trimethoxy-6-methylhomoisoflavanone under positive mode; (**E**): MS^1^ spectrum of ophiopojaponin C under negative mode; (**F**): MS^2^ spectra of ophiopojaponin C under negative mode; (**G**): MS^3^ spectra of ophiopojaponin C under negative mode; (**H**): The proposed fragmentation pathway of ophiopojaponin C under negative mode).

The MS^1^ fragment of l-borneol-7-*O*-[β-d-apiofuranosyl(1→6)]-β-d-glucopyranoside (peak 7, Rt = 3.988 min) was observed at *m*/*z* 447.2222 (Figure 2A), and the MS^2^ of the precursor 447.2222 fragment ion was at *m*/*z* 315.1796, produced by the loss of 132 Da from the [M − H]^−^ ion (Figure 2B).

Peak 7 is a known constituent of one of the Sheng-mai injectable formulations from *O. japonicus* [13,27] and no ambiguity arises from the MS data of YQFM. Homoisoflavonoids are a special class of flavonoids with their B-rings and C-rings connected at the C3 position and containing an additional CH_2_ group [1,28]. The quasi-molecular ions [M + H]^+^ and [M − H]^−^ were easily characterized under positive and negative mode. The MS^1^ fragments were subject to loss of the B-ring via the cleavage of C9-C1′ or C9-C3, and some examples exhibited successive loss of H_2_O under positive ion mode. For instance, the fragment ion of 5,2′-dihydroxy-7,8,4′-trimethoxy-6-methylhomoisoflavanone (peak 24, Rt = 9.433 min) under positive mode MS^1^ was [M + H]^+^ at *m*/*z* 375.1457 (Figure 2C), and the fragment ions from the precursor ion [M + H]^+^ 375.1457 were at *m*/*z* 357.1422, 251.0864, 236.0730 and 137.0694 (Figure 2D). Peak 24 is a known compound of one of *O. japonicus* [25]. According to the quasi-molecular ion and MS^n^ fragments, peak 54 may be a homoisoflavanone, but the specific structure cannot be confirmed without additional data.

**Figure 3:**
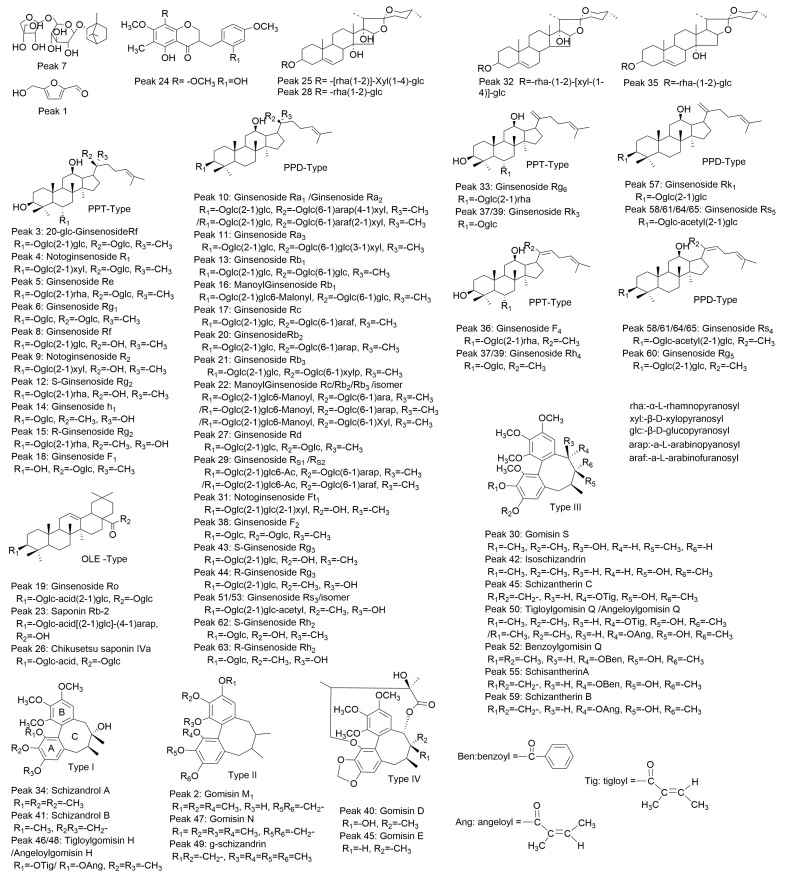
The structures of the sixty-five components identified from YiQiFuMai injection.

Steroidal saponins generated deprotonated ions [M − H]^−^ and typical solvent adduct ions [M + HCOO]^−^ under negative mode MS^1^, and the fragmentation patterns in MS^n^ from the precursor ions [M − H]^−^ or [M + HCOO]^−^ were mainly [M − H − saccharides]^−^ derived from simultaneous or successive losses of sugar moieties. The MS^1^ fragmentation of ophiopojaponin C (peak 25, Rt = 9.470 min) displayed an ion [M + HCOO]^−^ at *m*/*z* 931.4580 in negative mode (Figure 2E), and the MS^2^ of this precursor ion exhibited highly abundant ions [M − H]^−^ at *m*/*z* 885.4390 and [M − H − Xyl]^−^ at *m*/*z* 753.4087 (Figure 2F). The MS^3^ of precursor ion 885.4390 generated fragmentation ions at *m*/*z* 753.3965, 607.3377, and 445.2908 corresponding to [M − H − Xyl]^−^, [M − H − Xyl − Rha]^−^, and [M − H − Xyl − Rha − Glc]^−^, respectively (Figure 2G). The proposed fragmentation pathway of ophiopojaponin C is shown in Figure 2H. Peak 35 was a known constituent from *O. japonicus* as its main ion fragments were consistent with reported literature [1,26]. From the data, one L-borneol-7-*O*-[β-d-apiofuranosyl(1→6)]-β-d-glucopyranoside (peak 7), two homo- isoflavanones (peak 24 and peak 54) and four steroidal saponins (peaks 25, 28, 32 and 35) were identified and the corresponding structures are shown in Figure 3.

#### 2.2.2. Identification of Compounds in *P. ginseng*

Ginseng saponins are divided into three categories based on the parent skeleton, comprising the protopanaxadiol (PPD) type ginsenosides, the protopanaxatriol (PPT) type ginsenosides and the oleanolic acid (OLE) type [29,30]. In negative mode, the common fragmentation behavior of ginseng saponins was similar to that of steroidal saponins, with the MS^1^ spectra also exhibiting deprotonated ions [M − H]^−^ and typical solvent adduct ions [M + HCOO]^−^. Also, the common fragmentation behavior in the negative mode MS^n^ spectra was the successive or simultaneous loss of sugar units. For example, ginsenoside Rd (peak 27, Rt = 9.617) belongs to the PPD-type and exhibited [M − H]^−^ and [M + HCOO]^−^ ions at *m*/*z* 945.5297 and 991.5375, respectively (Figure S3A). The MS^2^ spectra of the precursor ion 991.5375 generated fragment ions [M − H]^−^ at *m*/*z* 945.5257 and [M − H − Glc]^−^ at *m*/*z* 783.4888 (Figure S3B). The MS^2^ spectra of precursor ion 945.5283 displayed highly abundant ions [M − H − Glc]^−^, [M − H − 2Glc]^−^, [M − H − 3Glc]^−^ and [M − H − 3Glc − C_6_H_12_]^−^ at *m*/*z* 783.4777, 621.4314, 459.3779 and 375.2875, respectively (Figure S3C). The precursor ion 783.4777 also gave a fragment ion [M − H − 3Glc − C_6_H_12_]^−^ at *m*/*z* 375.2858 in MS^3^ (Figure S3D). The fragmentation pathways for other ginsenoside types were similar to that of ginsenoside Rd (Figure S3E). Comparing the ion fragment information with reference standards and literature data [1,13,29,30,31,32,33], a total of forty-one ginsenosides were tentatively identified, including twenty-four PPD-type (peaks 10, 11, 13, 16, 17, 20, 21, 22, 27, 29, 31, 38, 43, 44, 51, 53, 57, 58, 60, 61, 62, 63, 64, 65), fourteen PPT-type (peaks 3, 4, 5, 6, 8, 9, 12, 14, 15, 18, 33, 36, 37, 39), and three OLE-type (peaks 19, 23, 26). Their chemical structures are shown in Figure 3.

#### 2.2.3. Identification of Compounds in *S. chinensis*

Lignans are comprised of A, B, and C rings, commonly containing different substituents, such as a hydroxy group on the C ring, a phenolic ester on an aromatic ring, an ester group and a lactone in an eleven-membered ring [32,34]. Characteristic positive ion mode lignan fragmentations are [M + Na]^+^, [M + H]^+^, [M + NH_4_]^+^, [M + H − H_2_O]^+^, [M + H − OCH_3_]^+^, [M + H − CH_2_O_2_]^+^, [M + H − C_5_H_10_]^+^, [M + H − C_5_H_8_O_2_]^+^, [M + H − C_7_H_6_O_2_]^+^ and [M + H − C_6_H_10_O_3_]^+^. Schizandrol A (peak 34, Rt = 11.065) generated [M + Na]^+^, [M + H]^+^ and [M + H − H_2_O]^+^ ions at *m*/*z* 455.2043, 433.2184 and 415.2085 in MS^1^, respectively (Figure S3F). The MS^2^ of precursor ion 433.2184 displayed consistent [M + H − H_2_O]^+^ fragment ions at *m*/*z* 415.2082 and a [M + H − H_2_O − OCH_3_]^+^ ion at *m*/*z* 384.1909 (Figure S3G), and the MS^3^ of precursor ion 415.2082 gave a consistent [M + H − H_2_O − OCH_3_]^+^ fragment ion at *m*/*z* 384.1915 (Figure S3H). The proposed fragmentation pathway of schizandrol A is shown in Figure S3J. From the data, a total of 16 lignans were confirmed by comparison of their mass spectrometric data with reference standards and literature data [1,13,32,35,36]. Their chemical structures are presented in Figure 3.

### 2.3. Quantitative Analysis of 21 Compounds in YQFM by UFLC-IT-TOF/MS

Although the use of HPLC-UV/ELSD for the determination of ginsenosides, ophiopogonins and lignans has been reported [10,14,37], the concentrations of some compounds, for example, ophiopogonins, were low in YQFM and the method was insufficiently sensitive or accurate for their detection. To solve this problem and simultaneously analyze multiple chemical constituents, UFLC-IT-TOF/MS was performed as a highly sensitive, selective and fast quantitative method.

#### 2.3.1. Method Validation

**Figure 4:**
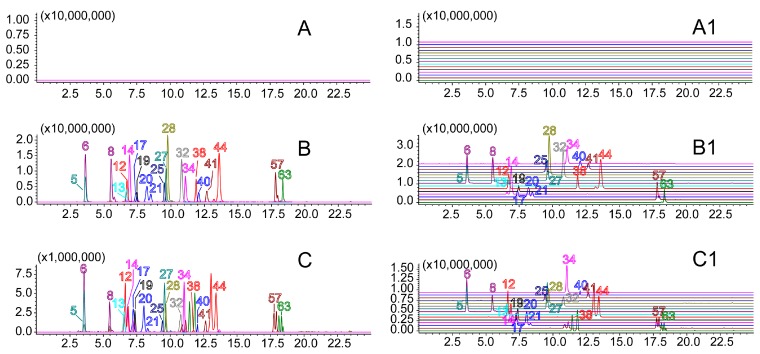
The extracted-ion chromatograms of 21 analytes determined by UFLC-IT-TOF/MS. ((**A**) & (**A1**): blank solution; (**B**) & (**B1**): reference solution; (**C**) & (**C1**): sample solution. 5: Re, 6: Rg_1_, 8: Rf, 12: S-Rg_2_, 13: Rb_1_, 14: Rh_1_, 17: Rc, 19: Ro, 20: Rb_2_, 21: Rb_3_, 25: ophiopojaponin C, 27: Rd, 28: ophiogenin 3-*O*-α-L-rhamnopyranosyl-(1→2)-β-d-glucopyranoside, 32: pennogenin-3-*O*-α-l-rhamnopyranosyl-(1→2)-β-d-xylopyranosyl-(1→4)-β-d-glucopyranoside, 34: schizandrol A, 38: F_2_, 40: gomisin D, 41: schizandrol B, 44: R-Rg_3_, 57: Rk_1_, 63: R-Rh_2_).

The specificity, linear range and sensitivity, precision, stability, reproducibility and accuracy of the developed method were validated. The specificity of the method showed a high resolution on the *m*/*z* axis using the proposed UFLC-IT-TOF/MS conditions with extracted ion in blank, mixed standard and sample solutions (Figure 4).

All calibration curves had good linearity with coefficients (*R*^2^) ≥0.9952 within the test ranges (Table S2). The limits of quantification (LOQ) of twenty-one compounds were 0.40–40.00 ng/mL (Table S2). These results (Table S3) indicated good precision for all analytes with relative standard deviations (RSDs) of intra- and inter-day precisions were less than 4.7% and 4.9%, respectively. The sample solution was found to be stable for 24 h at room temperature with an RSD <4.1% (Table S3). The RSDs for reproducibility of all analytes were <4.8% (Table S3). The established method had acceptable accuracy with spike recoveries at 91.8%–104.2% and RSDs <5.4% (Table S4). These method validation results indicated that the developed UFLC-IT-TOF/MS method was acceptable for the quantitative analysis of YQFM.

#### 2.3.2. Method Application for Quantification of 10 Batches of YQFM

Sample solutions of ten batches of YQFM were simultaneously determined using the validated UFLC-IT-TOF/MS method for twenty-one compounds. The summarized determination results (Table 1) indicated that the concentrations of twenty-one components varied in the ten batches.

The concentration ranges were 0.31–0.37 mg/g for the total three ophiopogonins, 6.66–9.24 mg/g for the total fifteen ginsenosides, and 0.13–0.17 mg/g for the total three lignans. These results demonstrated that the batch-to-batch variation was very low across these ten batches of YQFM, which may be a consequence of several factors. Firstly, all batches of samples were obtained from the same company in the same year, and their batch numbers were successive, suggesting that the raw material may have come from the same batch. Secondly, the YQFM raw materials have been collected from the same planting base for a long time. For example, *Ophiopogonis Radix* has always been sourced from Santai County (Sichuan Provence, China), to ensure that the quality of the raw material was consistent. Finally, the process technique is stable and strictly controlled during production.

Since the compositions of TCMs are complex, it is crucial to develop appropriate analytical methods to control consistency of the products. In this research, the concentration range of each component in different batches was consistent, indicating that the manufacturing processes were stable and reliable. The consistency of quality will ensure therapeutic efficacy and safety during YQFM treatment.

Saponins were the most abundant constituents in YQFM. A large number of studies have reported that ginsenosides, such as Rg_1_, Rb_1_, Rd, Rg_3_, Rh_1_, have beneficial effects on heart diseases [38,39,40,41,42], and some ophiopogonins have been reported to have potential effects on the cardiovascular system [18,43]. Moreover, schisandrin has been reported as a vascular endothelium protective component in YQFM [8,44]. The methods developed in this research were therefore successfully applied in the analysis of these main bioactive components in YQFM. Additionally, ophiopogonins were detected in YQFM for the first time in this work, and we plan to conduct further research on their biological function and mechanism.

## 3. Materials and Methods

### 3.1. Chemicals and Materials

Ten batches of YQFM (Z20060463; Batch. Nos: Y1–Y10) were obtained from Tianjin Tasly Pride Pharmaceutical Co. (Tianjin, China). The YQFM is composed of the ethanol extract (78 °C) of *Ginseng Radix et Rhizoma Rubra* from *Panax ginseng* C.A. Mey., and water extracts (100 °C) of *Ophiopogonis Radix* from *Ophiopogon japonicus* (L. f) Ker-Gawl. and *Schisandrae chinensis Fructus* from *Schisandra chinensis* (Turcz.) Baill combined in a ratio of 1:3:1.5. The yield of extracts was 23.64%, which were then subject to precision processing including multiple filtration, lyophilization, and aseptic packaging.

Authentic standards of ginsenosides Rb_1_, Re, Ro, Rb_2_, Rd, Rg_1_, Rf, Rc, Rb_3_, (20R)Rg_3_, Rk_1_, (20*S*)-Rg_2_, Rh_1_, (20*R*)-Rh_2_, F_2_, schizandrol A, gomisin D, schizandrol B and digoxin (internal standard, IS) were purchased from the National Institute for the Control of Pharmaceutical and Biological Products (Beijing, China). Ophiopojaponin C was obtained from Tianjin Shilan Technology Co. Ltd. (Tianjin, China). Ophiogenin 3-*O*-α-l-rhamnopyranosyl-(1→2)-β-d-glucopyranoside and pennogenin-3-*O*-α-l-rhamnopyranosyl-(1→2)-β-d-xylopyranosyl-(1→4)-β-d-glucopyranoside were isolated and purified from *Ophiopogonis Radix* in the authors’ laboratory and their structures were confirmed from their spectral data (MS, ^1^H- and ^13^C-NMR) according to the literature [27,45]. Their purities were >98% as determined by HPLC. Acetonitrile and methanol of LC-MS grade were purchased from Omni Chem. LLC (Morrisville, NC, USA). Formic acid was obtained from Sigma-Aldrich (St. Louis, MO, USA). Solid phase extraction (SPE) columns (Cleanert PS, 500 mg/6 mL) were obtained from Agela (Wilmington, DE, USA). Deionized water was prepared using a Milli-Q Ultrapure water system (Millipore, Bedford, MA, USA).

### 3.2. Standard Solutions and Sample Preparation

#### 3.2.1. Standard Solutions Preparation

Twenty-one reference compounds were accurately weighed and dissolved in methanol to prepare the mixed standard stock solution and stored at 4 °C until analysis. Internal standard stock solution was prepared in methanol at a concentration of 0.945 mg/mL and stored at 4 °C. Working solutions were prepared by appropriate dilution of the primary stock solutions with methanol. The final concentrations of IS was at 9.45 µg/mL in all working standard solutions.

#### 3.2.2. Sample Solution Preparation

##### 3.2.2.1. Sample Solutions for Qualitative Analysis

Accurately weighed YQFM (1.20 g) was dissolved in deionized water (5 mL), and then pretreated with SPE to remove interferences. Firstly, the SPE was activated with methanol (10 mL), followed by water (10 mL). The sample solution was then subjected to SPE, eluting sequentially with water, 20% methanol and 100% methanol (10 mL of each). The 100% methanol eluate was collected, evaporated to dryness and the residue was reconstituted with methanol to 5 mL in a volumetric flask. The solution was filtered through a 0.22 μm membrane, to provide the sample solution. Qualitative analysis by UFLC-IT-TOF/MS was performed using an injection volume of 5 μL.

##### 3.2.2.2. Sample Solutions for Quantitative Analysis

To ensure an appropriate concentration range for quantification, sample solutions were prepared at lower concentrations than those used for qualitative analysis. Accurately weighed YQFM (0.06 g) was dissolved in deionized water (5 mL), and then pretreated as described in Section 3.2.2.1. The 100% methanol eluate was collected in a 10 mL of volumetric flask. The final concentration of IS was 9.45 µg/mL in all sample solutions. The sample solution was filtered through a 0.22 μm membrane and the filtrate transferred to an autosampler vial for analysis.

### 3.3. UFLC-IT-TOF/MS Analysis Conditions

#### 3.3.1. Qualitative Analysis Conditions

Chromatographic experiments were conducted on a Shimadzu (Kyoto, Japan), UFLC system consisting of a CBM-20A controller, two LC-20AD binary pumps, an SIL-20AC autosampler, a CTO-20A column oven and a DGU-20A5 degasser. Chromatographic separation was performed on an ACQUITY UPLC HSS T3 column (100 mm × 2.1 mm, 1.8 µm) with the column temperature maintained at 30 °C. The mobile phase was composed of solvent A (ultrapure water containing 0.05%(*v*/*v*) formic acid) and solvent B (acetonitrile) and the gradient elution conditions were as follows: 0–2.5 min, 20%–30% B; 2.5–3 min, 30%–34% B; 3–8 min, 34%–37% B; 8–9 min, 37%–47% B; 9–16 min, 47%–50% B; 16–17 min, 50%–70% B; 17–18 min, 70%–85% B; 18–19 min, 85%–99% B; 19–22 min, 99% B; 22–25 min, 99%–20% B. The elution rate and injection volume were 0.4 mL/min and 5 µL, respectively.

The IT-TOF/MS mass spectrometer (Shimadzu) was equipped with an electrospray ionization (ESI) source. Ultrahigh purity argon was used as the collision gas and high purity nitrogen as the nebulizing gas. Positive and negative ion modes were performed at the same time. The following MS conditions were used: detector voltage, 1.65 kV; interface voltage 3.5 kV, curved desolvation line temperature 200 °C, heat block temperature 200 °C, nebulizing gas (N_2_) flow 1.5 L/min, drying gas pressure (N_2_) 72 kPa; ion trap pressure 1.9 × 10^−2^ Pa, TOF pressure 2.2 × 10^−4^ Pa, ion accumulation time 100 ms. Scan ranges were set at *m*/*z* 100–1500 for MS^1^, 50–1200 for MS^2^, 50–800 for MS^3^ and 50–400 for MS^4^. Accurate mass determination was corrected by calibration using the sodium trifluoroacetate clusters as reference, and the mass error was <5 mDa. Shimadzu’s Composition Formula Predictor software was used to predict the formulas of compounds.

#### 3.3.2. Quantitative Analysis Conditions

Chromatographic conditions were the same as those used for qualitative analysis (Section 3.3.1), except that mass detection was only scanned in MS^1^ over *m*/*z* 100–1500 in positive and negative ion modes. The observed ions were [M − H]^−^, [M + HCOO]^−^, [M + H]^+^ or [M + H − H_2_O]^+^ based on high response ions in the extracted-ion chromatograms.

### 3.4. Method Validation

To improve quantification precision and reproducibility, the specificity, linearity, sensitivity, precision, reproducibility, stability and accuracy of the established method were evaluated.

#### 3.4.1. Specificity

Extracted-ion chromatograms of sample, standard and blank solutions were compared to confirm whether the peak of each analyte was specific.

#### 3.4.2. Linear Range and Sensitivity

All stock solutions were diluted to appropriate concentrations for the construction of calibration curves with five concentrations analyzed in duplicate. The calibration curves were generated by determining the peak area ratios (analyte /internal standard) and the concentrations of twenty-one analytes. Sensitivity was determined by the limit of quantification (LOQ), defined as the analyte concentration producing a signal/noise ratio of about 10.

#### 3.4.3. Precision, Stability, Reproducibility and Accuracy

Variations in the intra- and inter-day data were employed to evaluate the precision of the developed method. Intra-day precision was performed with a mixed standard solution of twenty-one analytes, which was injected in six replicates in one day; inter-day variations were evaluated with the standard mixed solution which was injected in duplicates over three consecutive days. Intra- and inter-day precisions were expressed as the relative standard deviations (RSDs) of the peak area ratio (analyte /internal standard) and retention time of each analyte replicates. The stability test was performed by analysis of the sample solution at 0, 2, 4, 6, 12 and 24 h, and calculating the RSDs for peak area ratio (analyte /internal standard) and retention time of each analyte. For determination of reproducibility, six independently prepared YQFM sample solutions from the same batch were analyzed in triplicate and the RSD value calculated. Accuracy was calculated by the percentage recovery from a known added amount of each analyte in the sample. Known quantities of standards containing twenty-one analytes at three different concentration levels (80%, 100% and 120%) were added into a specific amount of YQFM in triplicate. The samples were prepared and analyzed using the methods described above and the spiked recoveries were calculated by the formula:

spiked recovery (%) = (found amount − original amount)/spiked amount × 100%(1)

### 3.5. Application to Analysis of Samples of YQFM

The quantitative analyses of ten batches of YQFM sample were performed using the developed UFLC-IT-TOF/MS as described in Section 3.3.

## 4. Conclusions

The efficacy and safety of TCM injection depends on the bioactive constituents and batch-to-batch consistency. It is therefore of great importance to establish appropriate analytical methods to determine the biologically active ingredients in TCMs. In the present study, a fast and reliable UFLC-IT-TOF/MS method was successfully employed for qualitative and quantitative analysis of multiple chemical constituents in YQFM. A total of sixty-five compounds were successfully separated and identified by comparing their retention times and MS spectra with those of authentic compounds and literature data. These included forty-two compounds from *P. ginseng*, sixteen compounds from *S. chinensis*, and seven compounds from *O. japonicus*, of which there were four steroidal saponins, one borneol pyranoside and two flavonoids. Moreover, the concentrations of twenty-one compounds were determined simultaneously, including the analysis of steroidal saponins from *O. japonicus* in YQFM, providing crucial information for the investigation of quality control methods and further substance-based mechanistic studies. This approach, which offers incredible advantages, including speed, simplicity and a reduction in solvent consumption, could become an important and widely used quality control technique for TCM.

## Figures and Tables

**Table 1:** The contents of 21 compounds in 10 batches of YQFM (μg/g, mean ± SD, *n* = 2).

Analyte	Y1	Y2	Y3	Y4	Y5	Y6	Y7	Y8	Y9	Y10
Peak 25	10.03 ± 0.12	10.54 ± 0.22	9.26 ± 0.40	8.35 ± 0.26	9.31 ± 0.33	9.22 ± 0.10	8.94 ± 0.20	9.81 ± 0.12	9.58 ± 0.21	9.93 ± 0.17
Peak 28	340.79 ± 9.34	338.47 ± 5.92	335.26 ± 14.29	291.53 ± 1.52	309.36 ± 2.44	316.55 ± 5.08	300.84 ± 4.62	316.60 ± 6.92	309.69 ± 2.47	337.38 ± 0.42
Peak 32	20.53 ± 0.74	20.16 ± 0.12	18.82 ± 0.10	14.27 ± 0.02	18.44 ± 0.86	18.80 ± 0.26	17.84 ± 0.20	20.36 ± 0.94	18.34 ± 0.78	21.90 ± 0.02
Re	239.22 ± 4.41	252.46 ± 2.39	260.48 ± 1.60	258.82 ± 3.84	269.16 ± 1.87	269.12 ± 5.50	264.25 ± 3.79	260.87 ± 0.35	260.10 ± 3.79	259.90 ± 3.60
Rg_1_	1655.22 ± 71.14	1718.39 ± 70.12	1763.12 ± 59.12	1726.97 ± 10.05	1822.21 ± 2.40	1798.42 ± 35.13	1744.87 ± 17.27	1596.24 ± 10.16	1646.69 ± 17.24	1880.85 ± 6.02
Rf	681.40 ± 15.78	608.42 ± 3.51	603.54 ± 16.76	751.55 ± 8.44	792.20 ± 38.74	810.92 ± 22.34	734.34 ± 34.89	809.52 ± 32.91	734.60 ± 33.41	878.60 ± 23.79
Rb_1_	1862.88 ± 34.38	1712.90 ± 25.32	1410.29 ± 65.97	1913.08 ± 93.66	2111.27 ± 80.02	2049.96 ± 3.82	1929.42 ± 71.45	2071.68 ± 29.32	2077.31 ± 77.55	2352.52 ± 37.43
S-Rg_2_	20.45 ± 0.31	19.60 ± 0.29	18.65 ± 0.16	20.83 ± 0.06	20.30 ± 0.28	20.53 ± 0.10	21.30 ± 0.85	20.49 ± 0.22	20.17 ± 0.19	20.65 ± 0.11
Ro	1538.77 ± 69.92	1474.66 ± 4.35	1313.99 ± 40.31	1615.63 ± 40.01	1573.38 ± 18.22	1632.99 ± 35.10	1606.87 ± 25.73	1657.41 ± 57.58	1623.26 ± 15.37	1673.61 ± 43.11
Rh_1_	473.68 ± 10.32	446.74 ± 2.02	388.87 ± 0.19	452.80 ± 3.69	427.09 ± 2.40	430.71 ± 9.74	429.85 ± 18.48	434.27 ± 4.07	417.29 ± 2.54	435.27 ± 1.74
Rc	84.83 ± 2.88	82.08 ± 2.00	80.59 ± 2.03	75.90 ± 0.13	79.84 ± 2.22	79.33 ± 0.53	78.03 ± 2.13	81.99 ± 3.66	76.47 ± 0.82	80.25 ± 1.38
Rb_2_	477.20 ± 2.00	459.21 ± 1.74	419.09 ± 9.67	478.33 ± 3.28	495.10 ± 3.62	509.51 ± 21.34	483.88 ± 13.24	516.15 ± 15.09	483.72 ± 7.12	521.33 ± 3.29
Rb_3_	98.25 ± 4.30	98.91 ± 0.80	89.71 ± 3.43	97.38 ± 0.58	103.03 ± 3.93	107.85 ± 0.86	101.33 ± 2.40	103.45 ± 4.03	97.51 ± 1.00	106.48 ± 2.91
Rd	672.80 ± 2.28	634.78 ± 3.03	601.17 ± 6.81	750.13 ± 29.17	760.54 ± 5.51	766.45 ± 1.01	727.08 ± 20.25	732.32 ± 23.51	728.89 ± 14.60	777.63 ± 6.88
F_2_	2.10 ± 0.06	2.12 ± 0.07	2.19 ± 0.05	2.55 ± 0.07	2.68 ± 0.01	2.78 ± 0.02	2.46 ± 0.07	2.62 ± 0.11	2.52 ± 0.08	2.77 ± 0.02
R-Rg_3_	143.24 ± 3.05	125.54 ± 1.43	101.18 ± 4.62	142.83 ± 4.49	143.42 ± 2.03	144.29 ± 3.34	143.62 ± 2.96	155.16 ± 2.29	144.93 ± 1.40	151.46 ± 1.20
Rk_1_	84.54 ± 1.54	72.62 ± 1.72	59.09 ± 3.16	86.94 ± 1.27	88.67 ± 1.98	88.81 ± 0.71	86.57 ± 1.10	94.12 ± 0.75	89.53 ± 4.26	94.05 ± 3.23
R-Rh_2_	0.94 ± 0.04	0.64 ± 0.02	0.71 ± 0.02	0.87 ± 0.01	0.98 ± 0.02	0.99 ± 0.01	0.92 ± 0.04	1.06 ± 0.04	1.04 ± 0.03	0.93 ± 0.02
Schizandrol A	129.08 ± 1.90	132.32 ± 1.70	123.19 ± 4.82	153.32 ± 2.64	155.45 ± 2.93	150.29 ± 2.66	142.81 ± 3.76	137.67 ± 4.66	142.85 ± 1.92	147.75 ± 1.46
Gomisin D	4.39 ± 0.13	4.90 ± 0.14	4.98 ± 0.02	5.56 ± 0.27	6.03 ± 0.29	5.67 ± 0.05	5.84 ± 0.04	5.77 ± 0.21	6.10 ± 0.04	5.69 ± 0.14
Schizandrol B	5.83 ± 0.20	6.52 ± 0.28	6.00 ± 0.25	7.03 ± 0.18	6.37 ± 0.10	6.82 ± 0.21	6.68 ± 0.32	6.55 ± 0.18	6.42 ± 0.22	6.31 ± 0.10

Note: Peak 25: ophiopojaponin C; Peak 28: ophiogenin 3-*O*-α-l-rhamnopyranosyl-(1→2)-β-d-glucopyranoside; Peak 32: pennogenin-3-*O*-α-l-rhamnopyranosyl-(1→2)-β-d-xylopyranosyl-(1→4)-β-d-glucopyranoside.

## Supplementary Materials

Supplementary materials can be accessed at: http://www.mdpi.com/1420-3049/21/5/640/s1.

## Abbreviations

YQFM	YiQiFuMai injection
UFLC-IT-TOF/MS	Ultra-fast liquid chromatography coupled with ion trap time-of-flight mass spectrometry
BPC	Base peak chromatogram
SPE	Solid phase extraction
TCM	Traditional Chinese Medicine
SMS	Sheng-mai San
