# Supplementary material for: Simultaneous Qualitative and Quantitative Analysis of Multiple Chemical Constituents in YiQiFuMai Injection by Ultra-Fast Liquid Chromatography Coupled with Ion Trap Time-of-Flight Mass Spectrometry
Source: Molecules. 2016 May 18;21(5):640. doi: 10.3390/molecules21050640 (PMC6272927; doi:10.3390/molecules21050640)
Supplement: Supplementary file 1 [file molecules-21-00640-s001.pdf]

Supplementary Materials: Simultaneous Qualitative and Quantitative Analysis of Multiple Chemical Constituents in YiQiFuMai Injection by Ultra-Fast Liquid Chromatography Coupled with Ion Trap Time-of-Flight Mass Spectrometry

Chunhua Liu, Aichun Ju, Dazheng Zhou, Dekun Li, Junping Kou, Boyang Yu and Jin Qi

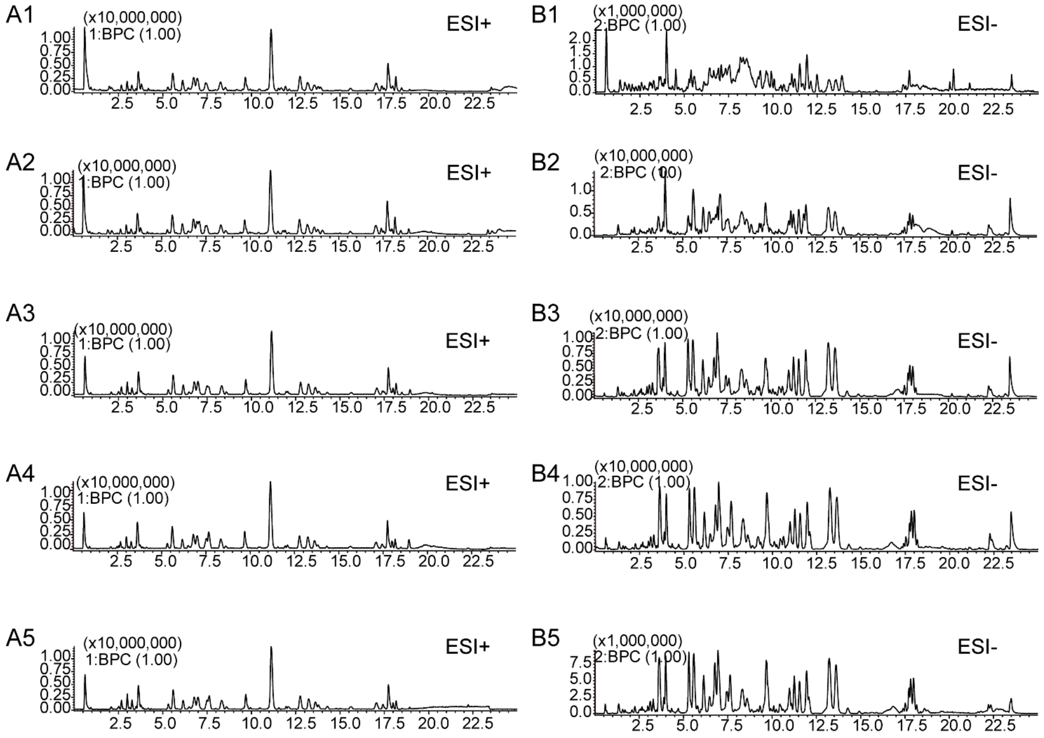

Figure S1. The BPC chromatograms of YQFM sample determined by UFLC-IT-TOF/MS under different mobile phase pH conditions and additives. ((**A1**,**B1**): H_2O-acetonitrile; (**A2**,**B2**): 0.05% (*v*/*v*) acetic acid H_2O-acetonitrile; (**A3**,**B3**): 0.05% (*v*/*v*) formic acid H_2O-acetonitrile; (**A4**,**B4**): 0.1% (*v*/*v*) formic acid H_2O-acetonitrile; (**A5**,**B5**): 0.05% (*v*/*v*) formic acid H_2O-0.05% (*v*/*v*) formic acid acetonitrile).

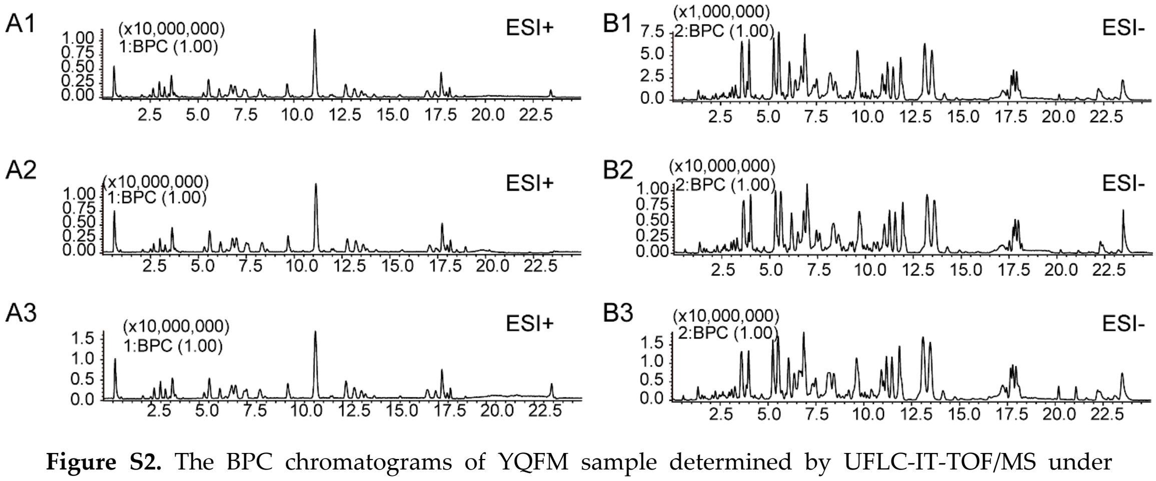

Figure S2. The BPC chromatograms of YQFM sample determined by UFLC-IT-TOF/MS under different accumulation times. ((**A1**,**B1**): Accumulation time was 30 ms; (**A2**,**B2**): Accumulation time was 50 ms; (**A3**,**B3**): Accumulation time was 100 ms).

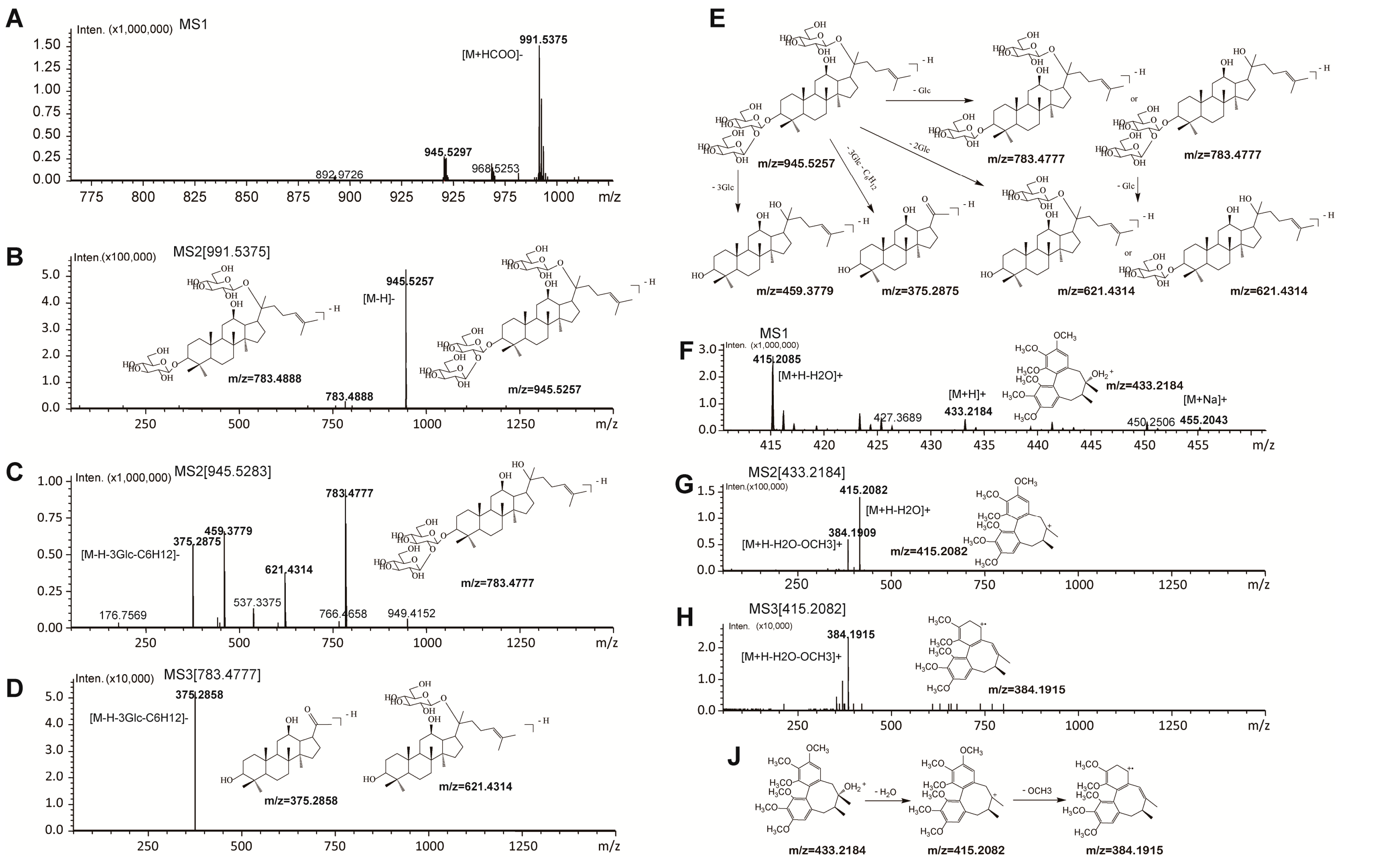

Figure S3. MS spectra and the proposed fragmentation pathways of ginsenoside Rd and schizandrol A. ((**A**): MS¹ spectra of ginsenoside Rd under negative mode; (**B**,**C**): MS² spectra of ginsenoside Rd under negative mode; (**D**): MS³ spectrum of ginsenoside Rd under negative mode; (**E**): The proposed fragmentation pathway of ginsenoside Rd under negative mode; (**F**): MS¹ spectra of schizandrol A under positive mode; (**G**): MS² spectra of schizandrol A under positive mode; (**H**): MS³ spectrum of schizandrol A under positive mode; (**J**): The proposed fragmentation pathway of schizandrol A under positive mode).

Table S1. Chemical information of 65 compounds identified from YQFM by UFLC-IT-TOF/MS.

No.	Rt (min)	Measured (m/z)	Predicted (m/z)	Diff (ppm)	Formula (M)	UFLC-ESI-MS^n (m/z)	Identification	Plant Material	Ref.
1	1.257	127.0396 $[M + H]^+$	127.0390	4.72	$C_6H_6O_3$		5-hydroxymethylfurfural	GS	[13]
2	2.980	387.1752 $[M + H]^+$	387.1802	−12.91	$C_{22}H_{26}O_6$	MS^2 [387.1752]:355.1219	Gomisin M_1	SF	[13,36]
3	3.108	1007.5337 $[M + COOH]^-$	1007.5432	−9.43	$C_{48}H_{82}O_{19}$	MS^2 [1007.5337]:961.5252,799.4669,638.4383; MS^3 [961.328]:475.3633	20-glc-GinsenosideRf	GS	[13]
4	3.273	977.5357 $[M + COOH]^-$	977.5327	2.97	$C_{47}H_{80}O_{18}$	MS^2 [977.5357]:931.5145	Notoginsenoside R_1	GS	[29,31]
5	3.603	991.5414 $[M + COOH]^-$	991.5483	−6.96	$C_{48}H_{82}O_{18}$	MS^2 [991.5414]:945.5365,784.4758	Ginsenoside Re *	GS	
6	3.662	845.4829 $[M + COOH]^-$	845.4904	−8.87	$C_{42}H_{72}O_{14}$	MS^2 [845.4829]:799.4757,637.4260,475.3754,391.2834; MS^3 [637.4260]:475.3683,391.2873	Ginsenoside Rg_1 *	GS	
7	3.988	447.2222 $[M - H]^-$	447.2236	−1.57	$C_{21}H_{36}O_{10}$	MS^2 [447.2222]:315.1796	L-borneol-7-*O*-[β-D-apiofuranosyl(1→6)]-β-D-glucopy-ranoside	OR	[13,27]
8	5.547	845.4840 $[M + COOH]^-$	845.4904	−7.57	$C_{42}H_{72}O_{14}$	MS^2 [845.4840]:799.4743,637.4268,475.3752; MS^3 [799.4769]:475.3681	Ginsenoside Rf *	GS	
9	6.078	769.4814 $[M - H]^-$	769.4744	9.10	$C_{41}H_{70}O_{13}$	MS^2 [769.4814]:637.4249,475.3728	Notoginsenoside R_2	GS	[32]
10	6.578	1209.6338 $[M - H]^-$	1209.6274	5.29	$C_{58}H_{98}O_{26}$	MS^2 [1209.6338]:1077.5718,945.5306,783.4827,621.4310,459.3807; MS^3 [1077.5715]:946.5246,459.3711	Ginsenoside Ra_1/Ginsenoside Ra_2	GS	[13]
11	6.628	1239.6312 $[M - H]^-$	1239.6379	−5.40	$C_{59}H_{100}O_{27}$		Ginsenoside Ra_3	GS	[13]
12	6.702	829.4926 $[M + COOH]^-$	829.4955	−3.50	$C_{42}H_{72}O_{13}$	MS^2 [829.4926]:783.4901,637.4224	S-Ginsenoside Rg_2 *	GS	
13	6.738	1107.5976 $[M - H]^-$	1077.5957	1.72	$C_{54}H_{92}O_{23}$	MS^2 [1107.5976]:945.5285,783.4772,621.4313,537.3311,459.3793	Ginsenoside Rb_1 *	GS	
14	6.880	683.4342 $[M + COOH]^-$	683.4376	−4.97	$C_{36}H_{62}O_9$	MS^2 [683.4342]:637.4305,475.3707	Ginsenoside h_1 *	GS	
15	6.922	829.4911 $[M + COOH]^-$	829.4955	−5.30	$C_{42}H_{72}O_{13}$	MS^2 [829.4884]:784.4798,638.4246,476.3792; MS^3 [783.4808]:537.7405,476.3791	R-Ginsenoside Rg_2	GS	[29,30]
16	6.995	1193.5782 $[M - H]^-$	1193.5961	−15.00	$C_{57}H_{94}O_{26}$	MS^2 [1193.5782]:1149.5936,1077.5735,945.5326,783.4777,621.4293,459.3900; MS^3 [1077.5371]:946.5159,459.3711	ManoylGinsenoside Rb_1/isomer	GS	[29,30]
17	7.307	1077.5800 $[M - H]^-$	1077.5851	−4.73	$C_{53}H_{90}O_{22}$	MS^2 [1077.5800]:945.5282,915.5194,783.4769,621.4325,459.3785,375.2866	Ginsenoside Rc *	GS	
18	7.367	683.4339 $[M + COOH]^-$	683.4376	−5.41	$C_{36}H_{62}O_9$	MS^2 [683.4339]:637.4248,475.3717; MS^3 [475.3717]:391.2832	Ginsenoside F_1	GS	[13,30]
19	7.472	955.4884 $[M - H]^-$	955.4908	−2.51	$C_{48}H_{76}O_{19}$	MS^2 [955.4884]:793.4294,631.3758,613.3682,569.3769,455.3480	Ginsenoside Ro *	GS	
20	8.168	1077.5829 $[M - H]^-$	1077.5851	−2.04	$C_{53}H_{90}O_{22}$	MS^2 [1077.5829]:945.5178,915.5167,783.4924,621.4297,537.3384,459.3842,375.2865	GinsenosideRb_2 *	GS	
21	8.443	1077.5763 $[M - H]^-$	1077.5851	−8.17	$C_{53}H_{90}O_{22}$	MS^2 [1077.5763]:945.5289,915.5194,783.4838,621.4310,537.3366,459.3782,375.2874	GinsenosideRb_3 *	GS	
22	8.590	1163.5809 $[M - H]^-$	1163.5855	−3.95	$C_{56}H_{92}O_{25}$	MS^2 [1163.5809]:1119.5832	ManoylGinsenoside Rc/Rb_2/Rb_3/isomer	GS	[13,29]
23	8.828	925.4767 $[M - H]^-$	925.4802	−3.78	$C_{47}H_{74}O_{18}$	MS^2 [925.4767]:763.4166,631.3802,613.3675,587.3938,569.3782,455.3450	Saponin Rb-2	GS	[29]
24	9.433	375.1457 $[M + H]^+$<br>373.1275 $[M-H]^-$	375.1438<br>373.1293	5.33<br>−4.82	$C_{20}H_{20}O_7$	MS^2 [375.1457]:357.1422,251.0864,137.0694; MS^2 [373.1275]:222.0528	5,2′-dihydroxy-7,8,4′-trimethoxy-6-methylhomoisoflavanone	OR	[25]
25	9.470	931.4580 $[M + COOH]^-$	931.4544	−7.30	$C_{44}H_{70}O_{18}$	MS^2 [931.4580]:885.4390, 753.4087; MS^3 [885.4453]:753.3965,607.3377,445.2908	Ophiopojaponin C *	OR	

26	9.562	793.4358 [M − H]⁻	793.4380	−2.77	$C_{42}H_{66}O_{14}$	MS² [793.4359]:631.3780,613.3703,569.3808,455.3478;<br>MS³ [631.3779]:455.3521	Chikusetsu saponin IVa	GS	[13]
27	9.617	991.5380 [M + COOH]⁻	991.5483	−10.39	$C_{48}H_{82}O_{18}$	MS² [991.5375]:945.5257,783.4888;<br>MS² [945.5483]:783.4777,621.4314,459.3779,375.2875;<br>MS³ [783.4777]:375.2858	Ginsenoside Rd *	GS	
28	9.727	799.4051 [M + COOH]⁻	799.4122	−8.88	$C_{39}H_{62}O_{14}$	MS² [799.4051]:753.4013,607.3435,446.2902	Ophiogenin 3-*O*-α-L-rhamnopyranosyl-(1→2)-β-D-glucopyranoside*	OR	
29	9.892	1119.5840 [M − H]⁻	1119.5957	−10.45	$C_{55}H_{92}O_{23}$	MS² [1119.5840]:1077.5735,945.5326,783.4777,621.4293,459.3900;<br>MS³ [1077.5731]:459.3711	Ginsenoside Rs_1/Rs_2	GS	[29]
30	9.928	455.2053 [M + Na]⁺	455.2040	2.86	$C_{24}H_{32}O_7$	MS² [433.2215]:415.2118,384.1952;<br>MS³ [415.2117]:384.1938	Gomisin S	SF	[1,36]
31	10.387	961.5311 [M + COOH]⁻	961.5378	−6.97	$C_{47}H_{80}O_{17}$	MS² [961.5311]:915.5170,783.4754,621.4288	Notoginsenoside Ft_1	GS	[33]
32	10.827	869.4431 [M − H]⁻<br>915.4505 [M + COOH]⁻	869.4540<br>915.4595	−12.54<br>−9.83	$C_{44}H_{70}O_{17}$	MS² [915.4505]:869.4403;<br>MS² [869.4431]:737.4030,591.3441	Pennogenin-3-*O*-α-L-rhamnopyranosyl-(1→2)-β-D-xylopyranosyl-(1→4)-β-D-glucopyranoside *	OR	
33	10.918	811.4798 [M + COOH]⁻	811.4849	−6.28	$C_{42}H_{70}O_{12}$	MS² [811.4798]:765.4752,620.4068	Ginsenoside Rg_6	GS	[13]
34	11.065	455.2043 [M + Na]⁺	455.2040	0.44	$C_{24}H_{32}O_7$	MS² [433.2184]:415.2082,384.1909;<br>MS³ [415.2082]:384.1915	Schizandrol A*	SF	
35	11.102	783.4154 [M + COOH]⁻	783.4172	−2.30	$C_{39}H_{62}O_{13}$	MS² [783.4154]:737.4046	PrazerigeninA3-*O*-α-L-rhamnopyranosyl-(1→2)-β-D-glucopyranoside	OR	[1,26]
36	11.193	811.4793 [M + COOH]⁻	811.4849	−6.90	$C_{42}H_{70}O_{12}$	MS² [811.4793]:765.4697,619.4047;<br>MS³ [765.4694]:619.4156	Ginsenoside F_4	GS	[1,13,33]
37	11.468	665.4218 [M + COOH]⁻	665.4270	−7.81	$C_{36}H_{60}O_8$	MS² [665.4218]:619.4135	Ginsenoside Rh_4/Rk_3	GS	[33]
38	11.762	829.4891 [M + COOH]⁻	829.4955	−7.72	$C_{42}H_{72}O_{13}$	MS² [829.4891]:783.4809,621.4319,459.3840;<br>MS³ [783.4824]:459.3685,375.2851:	Ginsenoside F_2 *	GS	
39	11.872	665.4231 [M + COOH]⁻	665.4270	−5.86	$C_{36}H_{60}O_8$	MS² [665.4231]:619.4047,457.3658	GinsenosideRk_3/Rh_4	GS	[33]
40	12.073	548.2487 [M + NH₄]⁺	548.2490	−0.55	$C_{28}H_{34}O_{10}$	MS² [548.2487]:531.2139,485.2204,401.1361,383.1527; MS³ [401.1623]:341.1198	Gomisin D *	SF	
41	12.678	439.1732 [M + Na]⁺	439.1727	1.14	$C_{23}H_{28}O_7$	MS² [417.1609]: 399.1793,341.1446	Schizandrol B *	SF	
42	12.843	455.1983 [M + Na]⁺	455.2040	−12.52	$C_{24}H_{32}O_7$	MS² [433.1931]:415.2130	Isoschizandrin	SF	[13]
43	13.100	829.4914 [M + COOH]⁻	829.4955	−4.94	$C_{42}H_{72}O_{13}$	MS² [829.4914]:784.4810,621.4256,460.3715;<br>MS³ [783.4788]:622.4254,375.2851	S-Ginsenoside Rg_3	GS	[13,33]
44	13.467	829.4894 [M + COOH]⁻	829.4955	−7.35	$C_{42}H_{72}O_{13}$	MS² [829.4894]:784.4865,622.4254;<br>MS³ [783.4876]:337.5247	R-Ginsenoside Rg_3 *	GS	
45	13.668	515.2260 [M + H]⁺	515.2276	−3.11	$C_{28}H_{34}O_9$	MS² [515.2260]:385.1691,355.1556;<br>MS³ [385.1694]:338.6956	Gomisin E/Schizantherin C	SF	[34]
46	14.218	523.2295 [M + Na]⁺	523.2303	−1.34	$C_{28}H_{36}O_8$	MS² [501.2436]:483.2329,431.2043,401.1919	Tigloylgomisin H/Angeloylgomisin H	SF	[32]
47	14.695	401.1928 [M + H]⁺<br>423.1728 [M + Na]⁺	401.1959<br>423.1778	−7.73<br>−11.82	$C_{23}H_{28}O_6$		Gomisin N	SF	[36]
48	15.488	523.2261 [M + Na]⁺	523.2303	−7.84	$C_{28}H_{36}O_8$	MS² [501.2453]:483.2363,431.2044,401.1940;<br>MS³ [401.1941]:337.1437	AngeloylgomisinH/Tigloylgomisin H	SF	[1,13,32]
49	15.520	401.1934 [M + H]⁺	401.1959	−6.23	$C_{23}H_{28}O_6$	MS² [401.1934]:386.1580,370.1729,355.1518	g-schizandrin	SF	[13]

50	15.575	548.2848 [M + NH4]$^+$	548.2854	−1.09	$C_{29}H_{38}O_9$	MS^2 [548.2848]:431.2058,387.1864; MS^2 [531.2532]:483.2364	TigloylgomisinQ/Angeloylgomisin Q	SF	[32]
51	16.547	871.5066 [M + COOH]$^-$	871.5061	0.57	$C_{44}H_{74}O_{14}$	MS^2 [871.5066]:825.4936,765.4619; MS^3 [825.4936]:783.4733,621.4288	Ginsenoside Rs$_3$/isomer	GS	[33]
52	16.932	548.2814 [M + NH4]$^+$	548.2854	−7.30	$C_{29}H_{38}O_9$	MS^2 [548.2814]:431.2100; MS^3 [431.2100]:387.1934,356.1578	Benzoylgomisin Q	SF	[1,36]
53	17.133	871.5038 [M + COOH]$^-$	871.5061	−2.64	$C_{44}H_{74}O_{14}$	MS^2 [871.5038]:825.5321; MS^3 [825.4936]:783.4813,765.4631,621.4288,459.3711	Ginsenoside Rs$_3$/isomer	GS	[1,33]
54	17.353	343.1535 [M + H]$^+$	343.1540	−2.62	$C_{20}H_{22}O_5$	MS^2 [343.1535]:221,0837,121.0630; MS^2 [341.1388]:299.0859	Homoisoflavanone	OR	
		341.1388 [M − H]$^-$	341.1394	−6.45					
55	17.445	554.2324 [M + NH$_4$]$^+$	554.2232	16.6	$C_{26}H_{32}O_{12}$	MS^2 [537.3450]:415.1837,355.2830,337.2751	SchisantherinA/GomisinG	SF	[1,32]
56	17.573	532.2495 [M + NH$_4$]$^+$	532.2541	−8.67	$C_{28}H_{34}O_9$	MS^2 [532.2495]:415.1763	Gomisin F	SF	[1,32]
57	17.775	811.4779 [M + COOH]$^-$	811.4849	−8.63	$C_{42}H_{70}O_{12}$	MS^2 [811.4779]:765.4708	Ginsenoside Rk$_1$ *	GS	
58	17.867	853.4890 [M + COOH]$^-$	853.4955	−7.62	$C_{44}H_{72}O_{13}$	MS^2 [853.4890]:807.4766,765.4784	Ginsenoside Rs$_4$/Rs$_5$	GS	[33]
59	17.885	532.2552 [M + NH$_4$]$^+$	532.2541	2.07	$C_{28}H_{34}O_9$	MS^2 [532.2552]:415.1721,385.1599,371.1461	Schizantherin B	SF	[1,34]
60	17.940	811.4800 [M + COOH]$^-$	811.4849	−6.04	$C_{42}H_{70}O_{12}$	MS^2 [811.4800]:765.4736	Ginsenoside Rg$_5$	GS	[1,13,33]
61	18.032	853.4912 [M + COOH]$^-$	853.4955	−5.04	$C_{44}H_{72}O_{13}$	MS^2 [853.4912]:807.4880,765.4731	Ginsenoside Rs$_4$/Rs$_5$	GS	[33]
62	18.142	667.4317 [M + COOH]$^-$	667.4215	15.28	$C_{39}H_{58}O_6$	MS^2 [667.4317]: 621.4230,459.3860	S-Ginsenoside Rh$_2$	GS	[30]
63	18.343	667.4309 [M + COOH]$^-$	667.4215	14.08	$C_{39}H_{58}O_6$		R-Ginsenoside Rh$_2$ *	GS	
64	18.655	853.4908 [M + COOH]$^-$	853.4955	−5.51	$C_{44}H_{72}O_{13}$	MS^2 [853.4908]:807.4784,765.4693	Ginsenoside Rs$_4$/Rs$_5$	GS	[33]
65	18.765	853.4961 [M + COOH]$^-$	853.4955	0.70	$C_{44}H_{72}O_{13}$	MS^2 [853.4961]:807.4887	Ginsenoside Rs$_4$/Rs$_5$	GS	[33]

Note: GS: Ginseng Radix et Rhizoma Rubra; SF: Schisandrae chinensis Fructus; OR: Ophiopogonis Radix. * Compared with authentic compounds.

Table S2. The retention times, observed *m*/*z*, limits of quantification (LOQ) and regression equations of 21 analytes.

Peak No.	Compounds	Retention Time (min)	Observed m/z	LOQ (ng/mL)	Dynamicrange (ng/mL)	Calibration Curves Equation	R^2
25	Peak 25	9.49	931.4445 $[M + HCOO]^-$	1.50	18.75–300	y =0.0214x + 0.178	0.9981
28	Peak 28	9.73	799.4024 $[M + HCOO]^-$	1.42	178–2848	y = 0.0115x + 2.011	0.9967
32	Peak 32	10.78	915.4464 $[M + HCOO]^-$	1.25	155.75–2492	y = 0.0097x + 1.325	0.9984
5	Re	3.58	991.5334 $[M + HCOO]^-$	1.20	150–2400	y = 0.006x + 0.5387	0.9970
6	Rg_1	3.59	845.4834 $[M + HCOO]^-$	1.10	137.5–2200	y = 0.0107x + 0.6561	0.9964
8	Rf	5.52	845.4834 $[M + HCOO]^-$	1.20	150–2400	y = 0.0079x + 0.6719	0.9981
13	Rb_1	6.64	1107.5890 $[M - H]^-$	2.60	325–5200	y = 0.0016x − 0.216	0.9999
12	S-Rg_2	6.70	829.4871 $[M + HCOO]^-$	0.40	50–800	y = 0.0165x + 0.3454	0.9980
19	Ro	7.55	955.9874 $[M - H]^-$	40.00	100–4000	y = 0.0016x − 0.2344	0.9993
14	Rh_1	6.89	683.4297 $[M + HCOO]^-$	4.00	50–800	y =0.0232x − 0.0511	1.00
17	Rc	7.31	1077.5740 $[M - H]^-$	12.50	156–2500	y = 0.0021x − 0.2315	0.9989
20	Rb_2	8.17	1077.5740 $[M - H]^-$	2.50	312–5000	y = 0.0019x − 0.5149	0.9977
21	Rb_3	8.46	1077.5740 $[M - H]^-$	7.00	87.5–7000	y = 0.0019x − 0.1633	0.9998
27	Rd	9.63	991.5334 $[M + HCOO]^-$	1.00	125–2000	y = 0.0083x + 0.6136	0.9952
38	F_2	11.76	829.4871 $[M + HCOO]^-$	3.00	37.5–600	y = 0.0129x − 0.0919	1.00
44	R-Rg_3	13.55	829.4871 $[M + HCOO]^-$	0.80	100–1600	y = 0.0199x + 0.8986	0.9984
57	Rk_1	17.80	811.4755 $[M + HCOO]^-$	4.00	50–800	y = 0.0142x + 0.5358	0.9955
63	R-Rh_2	18.35	667.4368 $[M + HCOO]^-$	2.00	25–400	y = 0.017x − 0.0315	0.9999
34	Schizandrol A	11.07	415.2109 $[M + H\text{-}H2O]^+$	1.10	137.5–2200	y = 0.0071x + 0.771	0.9970
40	Gomisin D	12.06	531.2226 $[M + H]^+$	10.00	12.5–1000	y = 0.0015x − 0.0074	0.9992
41	Schizandrol B	12.66	399.1779 $[M + H - H_2O]^+$	4.00	50–800	y = 0.0081x + 0.0724	0.9986

Note: Peak 25: ophiopojaponin C; Peak 28: ophiogenin 3-*O*-α-L-rhamnopyranosyl-(1→2)-β-D-glucopyranoside; Peak 32: pennogenin-3-*O*-α-L-rhamnopyranosyl-(1→2)-β-D-xylopyranosyl-(1→4)-β-D-glucopyranoside; y was the Ratio of peak area analytes by IS (peak area of analytes/peak area of internal standard), x was the concentration of compounds (ng/mL).

Table S3. Precision, stability and reproducibility of 21 compounds.

Peak No.	Analytes	Precision				Stability (n = 6)		Reproducibility (n = 6)	
		Intra-Day (n = 6)		Inter-Day (n = 3)					
		Rt RSD (%)	Area Ratio by IS RSD (%)	Rt RSD (%)	Area Ratio by IS RSD (%)	Rt RSD (%)	Area Ratio by IS RSD (%)	Rt RSD (%)	Concentration RSD (%)
25	Peak 25	0.13	3.54	0.17	3.08	0.23	0.86	0.25	4.38
28	Peak 28	0.09	0.88	0.09	0.92	0.17	2.25	0.18	3.00
32	Peak 32	0.06	1.67	0.63	0.60	0.14	3.17	0.15	2.44
5	Re	0.29	0.96	0.31	1.21	0.47	1.78	0.34	2.20
6	Rg_1	0.28	1.42	0.30	2.55	0.32	1.12	0.36	2.62
8	Rf	0.15	1.89	0.22	3.77	0.27	0.90	0.37	3.23
13	Rb_1	0.29	2.82	0.32	4.33	0.44	3.14	0.44	3.23
12	S-Rg_2	0.16	1.55	0.25	2.87	0.35	2.39	0.38	4.79
19	Ro	0.19	3.30	0.30	4.45	0.49	4.14	0.47	4.11
14	Rh_1	0.18	2.78	0.25	2.51	0.36	2.07	0.36	3.78
17	Rc	0.25	2.25	0.33	2.59	0.52	0.82	0.5	2.26
20	Rb_2	0.10	4.13	0.34	3.32	0.58	1.72	0.56	3.81
21	Rb_3	0.25	2.94	0.35	4.86	0.53	2.45	0.58	3.70
27	Rd	0.08	1.81	0.11	2.58	0.15	2.37	0.17	2.93
38	F_2	0.09	3.88	0.10	4.56	0.14	1.93	0.15	4.36
44	R-Rg_3	0.10	2.11	0.11	4.45	0.21	1.56	0.19	4.23
57	Rk_1	0.04	3.27	0.04	2.67	0.05	2.74	0.06	3.78
63	R-Rh_2	0.02	3.25	0.03	3.28	0.05	2.17	0.05	4.14
34	Schizandrol A	0.05	2.00	0.09	3.18	0.14	1.34	0.14	3.82
40	Gomisin D	0.13	4.66	0.14	4.37	0.19	3.18	0.13	4.30
41	Schizandrol B	0.04	2.57	0.08	4.58	0.17	1.94	0.18	4.40

Note: Peak 25: ophiopojaponin C; Peak 28: ophiogenin 3-*O*-α-L-rhamnopyranosyl-(1→2)-β-D-glucopyranoside; Peak 32: pennogenin-3-*O*-α-L-rhamnopyranosyl-(1→2)-β-D-xylopyranosyl-(1→4)-β-D-glucopyranoside. Rt: Retention times.

Table S4. Recovery test.

Analytes	Recovery														
	Low (*n* = 3)					Medium (*n* = 3)					High (*n* = 3)				
	Original (ng)	Spiked (ng)	Found (ng)	Recovery (%)	RSD (%)	Original (ng)	Spiked (ng)	Found (ng)	Recovery (%)	RSD (%)	Original (ng)	Spiked (ng)	Found (ng)	Recovery (%)	RSD (%)
Peak 25	455.32 ± 6.82	360	810.28 ± 23.02	96.17 ± 3.07	3.12	469.0 ± 10.58	450	893.40 ± 10.28	94.31 ± 1.52	1.63	453.12 ± 3.52	562.5	991.84 ± 19.97	95.77 ± 3.74	3.90
Peak 28	851.70 ± 14.18	640	1512.474 ± 6.78	103.12 ± 3.14	3.04	866.71 ± 23.64	854.4	1727.47 ± 46.07	100.74 ± 4.26	4.23	841.69 ± 5.01	996.8	1844.25 ± 59.46	100.25 ± 4.93	4.92
Peak 32	1296.81 ± 19.02	996.8	2274.82 ± 20.28	98.11 ± 3.63	3.70	1318.08 ± 23.65	1246	2480.84 ± 49.87	93.32 ± 2.64	2.83	1311.63 ± 54.02	1557.5	2808.53 ± 113.52	96.11 ± 4.84	5.04
Re	61,574.3 ± 789.78	47,570	109,606.2 ± 1734.68	100.97 ± 2.59	2.56	63,111.65 ± 1528.19	58,930	118,927.6 ± 1792.88	94.23 ± 1.85	1.96	61,993.17 ± 2170.27	68,600	129,012.4 ± 4752.02	97.67 ± 2.64	2.70
Rg_1	13,380.66 ± 175.66	10,440	23,588.93 ± 436.26	97.78 ± 3.35	3.43	13,763.09 ± 324.60	12,760	26,642.01 ± 510.85	100.932 ± 2.27	2.25	13,513.86 ± 473.10	15,370	27,968.99 ± 891.12	94.05 ± 3.07	3.27
Rf	20,880.23 ± 267.82	16,100	36,407.46 ± 446.06	96.44 ± 4.01	4.16	21,486.47 ± 470.12	20,240	41,564.91 ± 910.46	99.20 ± 3.46	3.49	21,147.61 ± 778.86	24,380	45,419.3 ± 1531.02	99.56 ± 4.82	4.84
Rb_1	90,747.25 ± 1191.29	61,200	152,502.3 ± 2829.53	99.04 ± 3.74	3.78	93,719.71 ± 2381.79	84,660	179,477.90 ± 4526.64	101.30 ± 5.03	4.97	91,650.65 ± 3208.53	100,400	190,073 ± 5368.09	98.03 ± 3.60	3.68
S-Rg_2	4508.96 ± 53.02	3430	7865.32 ± 107.95	102.13 ± 1.84	1.80	4698.20 ± 124.42	4165	8627.81 ± 292.01	94.35 ± 5.10	5.40	4554.83 ± 159.46	4900	9054.46 ± 206.09	91.83 ± 1.34	1.46
Ro	38,338.13 ± 503.29	28,810	67,042.60 ± 1565.11	99.63 ± 4.64	4.66	39,396.92 ± 886.94	35,690	74,683.42 ± 1257.38	98.87 ± 1.70	1.72	38,781.34 ± 1529.95	43,750	83,971.41 ± 600.90	103.28 ± 1.94	1.88
Rh_1	3414.69 ± 44.83	2660	6003.54 ± 121.89	97.32 ± 3.90	4.01	3499.24 ± 76.21	3230	6688.24 ± 208.83	98.73 ± 4.69	4.75	3392.48 ± 26.34	3800	7091.45 ± 135.08	97.34 ± 4.10	4.22
Rc	19,574.34 ± 261.26	16,200	35,990.73 ± 428.19	101.34 ± 2.52	2.48	20,299.99 ± 404.52	19,800	39,161.30 ± 1085.64	95.26 ± 4.21	4.42	19,962.05 ± 857.93	23,850	42,567.96 ± 898.76	94.78 ± 4.77	5.03
Rb_2	44,240.98 ± 580.78	34,800	78,973.77 ± 1319.48	99.81 ± 4.45	4.46	45,758.77 ± 1192.40	48,140	95,261.87 ± 1357.86	102.83 ± 3.69	3.59	45,503.81 ± 1546.39	53,000	96,580.68 ± 1966.61	98.40 ± 3.96	4.02
Rb_3	6108.98 ± 91.17	5250	11,407.95 ± 103.99	100.93 ± 1.98	1.96	6301.07 ± 164.11	6600	12,751.25 ± 403.48	97.73 ± 4.12	4.21	6183.30 ± 243.93	7950	14,220.15 ± 232.82	101.09 ± 1.77	1.75
Rd	31,870.13 ± 418.38	26,130	57,612.79 ± 1215.48	98.52 ± 3.76	3.82	32,881.88 ± 924.34	32,370	63,834.24 ± 1472.14	95.62 ± 4.01	4.19	32,366.50 ± 1203.83	36,750	66,645.93 ± 3556.12	93.19 ± 2.94	3.15
F_2	377.92 ± 5.66	294.5	679.16 ± 14.09	102.29 ± 4.40	4.30	388.68 ± 11.64	372	753.87 ± 14.64	98.99 ± 5.06	5.16	376.56 ± 2.92	449.5	826.68 ± 22.01	100.14 ± 5.00	4.99
R-Rg_3	7292.36 ± 30.53	6120	13,400.23 ± 230.11	99.80 ± 3.30	3.30	7670.78 ± 148.58	7480	15,201.8 ± 456.86	100.68 ± 4.59	4.56	7430.32 ± 247.79	9061	16,048.14 ± 331.61	95.11 ± 1.54	1.62
Rk_1	5835.68 ± 73.83	4900	10,751.99 ± 175.91	100.33 ± 4.19	4.18	6000.09 ± 179.70	6160	12,046.14 ± 211.38	98.15 ± 1.07	1.09	5813.45 ± 53.31	7462	13,140.45 ± 224.81	98.19 ± 3.05	3.11
R-Rh_2	138.46 ± 1.84	90	227.77 ± 4.78	99.23 ± 5.29	5.32	142.37 ± 2.83	140	288.19 ± 8.61	104.16 ± 5.05	4.85	135.70 ± 0.74	145	281.34 ± 4.07	100.45 ± 3.22	3.21
Schizandrol A	9595.70 ± 135.19	8280	17,664.42 ± 441.30	97.45 ± 3.76	3.86	9814.27 ± 231.47	10,120	19,670.08 ± 672.51	96.10 ± 3.72	3.87	9680.60 ± 366.63	12,190	21,833.98 ± 474.73	99.70 ± 3.01	3.02
Gomisin D	609.32 ± 8.34	432	1034.94 ± 15.43	98.52 ± 2.23	2.27	628.80 ± 14.83	596	1220.41 ± 27.66	99.26 ± 2.73	2.76	626.90 ± 23.89	696	1302.7 ± 15.61	97.10 ± 2.62	2.70
Schizandrol B	662.67 ± 7.80	487	1144.37 ± 1608	99.11 ± 2.04	2.06	677.23 ± 14.34	648	1296.41 ± 11.13	95.55 ± 1.75	1.83	669.42 ± 23.72	783	1454.08 ± 37.72	100.21 ± 2.76	2.76

Note: Peak 25: ophiopojaponin C; Peak 28: ophiogenin 3-*O*-α-L-rhamnopyranosyl-(1→2)-β-D-glucopyranoside; Peak 32: pennogenin-3-*O*-α-L-rhamnopyranosyl-(1→2)-β-D-xylopyranosyl-(1→4)-β-D-glucopyranoside.
